# Worldwide productivity and research trend of publications concerning glioma-associated macrophage/microglia: A bibliometric study

**DOI:** 10.3389/fneur.2022.1047162

**Published:** 2022-12-08

**Authors:** Yu-yang Liu, Ren-qi Yao, Li-yan Long, Yu-xiao Liu, Bing-Yan Tao, Hong-yu Liu, Jia-lin Liu, Ze Li, Ling Chen, Yong-ming Yao

**Affiliations:** ^1^Translational Medicine Research Center, Medical Innovation Research Division and Fourth Medical Center of the Chinese PLA General Hospital, Beijing, China; ^2^Medical School of Chinese PLA, Beijing, China; ^3^Department of Neurosurgery, Chinese PLA General Hospital, Beijing, China; ^4^Department of Burn Surgery, The First Affiliated Hospital of Naval Medical University, Shanghai, China; ^5^Library, Medical School of Chinese PLA, Beijing, China

**Keywords:** bibliometrics, glioma, glioblastoma, macrophage, microglia

## Abstract

Glioma-associated macrophage/microglia (GAM) represents a key player in shaping a unique glioma ecosystem to facilitate tumor progression and therapeutic resistance. Numerous studies have been published concerning GAM, but no relevant bibliometric study has been performed yet. Our bibliometric study aimed to comprehensively summarize and analyze the global scientific output, research hotspots, and trendy topics of publications on GAM over time. Data on publications on GAM were collected using the Web of Science (WoS). The search date was 16 January 2022, and the publications were collected from 2002 to 2021. Totally, 1,224 articles and reviews were incorporated and analyzed in the current study. It showed that the annual publications concerning GAM kept increasing over the past 20 years. The United States had the largest number of publications and total citations. Holland, Kettenmann, and Gutmann were the top three authors in terms of citation frequency. Neuro-oncology represented the most influential journal in GAM studies, with the highest H-index, total citations, and publication numbers. The paper published by Hambardzumyan in 2016 had the highest local citations. Additionally, the analysis of keywords implied that “prognosis,” “tumor microenvironment,” and “immunotherapy” might become research hotspots. Furthermore, trendy topics in GAM studies suggested that “immune infiltration,” “immune microenvironment,” “bioinformatics,” “prognosis,” and “immunotherapy” deserved additional attention. In conclusion, this bibliometric study comprehensively analyzed the publication trend of GAM studies for the past 20 years, in which the research hotspots and trendy topics were also uncovered. This information offered scholars critical references for conducting in-depth studies on GAM in the future.

## Introduction

Glioma has been considered to be the majority of primary intracranial malignancies, with poor prognosis, high recurrence, and mortality rate. Although great progress has been made in advanced multimodality regimens, the clinical outcomes of patients with glioma remained dismal (1, 2). In line with the latest World Health Organization (WHO) definition, adult gliomas primarily include tumors ranging from WHO grade II to IV (3). Glioblastoma (GBM, WHO grade IV), the most life-threatening subtype of glioma, is extremely resistant to conventional therapies, with a median survival of 14 months (4).

It has been well-accepted that the tumor microenvironment (TME) plays a pivotal role in sustaining the malignant proliferation and progression of GBM (5). The TME of GBM consists of various components, including endothelial cells, vascular pericytes, cancer-associated fibroblasts, infiltrating immune cells, and extracellular matrix (6–8). Among them, the glioma-associated macrophage/microglia (GAM) represent the most abundant cell type in the TME, comprising as many as 30–50% of all cells in human GBM (9). Of note, 85% of GAM are infiltrating macrophages/monocyte, while the remaining 15% are resident microglia (10). Recent studies have demonstrated that GAM could be divided into two major subpopulations: the M2 macrophage/microglia (tumor-supportive subtype) and M1 macrophage/microglia (tumor-suppressive subtype). The M2 subtype has an intimate association with the immunosuppressive status of TME in GBM (11, 12). Notably, the majority of GAM in GBM exhibit M2-like properties, with potent immunosuppressive capacity (13). Furthermore, the GAM's density has been demonstrated to be positively correlated with the glioma grade and poor prognosis among patients with glioma (14, 15). Despite the significant role of GAM in GBM, the mechanisms underlying tumor-supportive functions of GAM have not been established yet.

Bibliometrics represents a branch of library science that uses mathematical and statistical measurements to analyze publications quantitatively and qualitatively (16). Based on multidimensional analyses, the bibliometric methodology can depict the trend of published literature and investigate the patterns of publications in a specific research field (17). Additionally, it can facilitate researchers in grasping the key research focuses and predicting future tendencies (18). At present, bibliometric has been generally used in various fields, including orthopedics, neuro-oncology, infectious disease, and others (18–22). Nevertheless, to the best of our knowledge, bibliometrics-based study on GAM remains a virgin land. Correspondingly, it is necessary to conduct an integrated analysis of the present status, research hotspots, and future tendencies of publications concerning GAM.

## Materials and methods

### Data source and search strategy

We searched the Web of Science (WoS) on 16 January 2022 to collect GAM-related studies between 2002 and 2021. The database source was limited to Science Citation Index Expanded. The search strategy was presented as follows: TS = glioma-associated macrophage OR TS = glioma AND (tumor-associated macrophage OR tumor-associated microglia OR TAM) OR TS = glioblastoma-associated macrophage OR TS = GBM-associated macrophage OR TS = (glioblastoma OR GBM) AND (tumor-associated macrophage OR tumor-associated microglia OR TAM).

### Eligibility criteria and data collection

The publication types were limited to “article” and “review,” which were written in English. The meeting abstract, editorial material, book chapter, letter, and others were excluded. Duplicate and inaccurate raw data were removed by the analyzing tool automatically. Eventually, a total of 1,224 publications were incorporated and analyzed in the current study. All the information, including titles, authors, affiliations, sources, countries, keywords, publication year, and references, was retrieved for subsequent bibliometric analysis.

### Bibliometric analyses and visualization

The R software (version 4.1.2) and the “BiblioShiny” package were used to construct the basic analysis of all enrolled publications. “BiblioShiny” is the tool under the package that is designed for non-coders to provide methods for complete bibliometric analysis. It enables the generation of multiple results in the form of tables and graphs, which are not common in other software (21). To optimize the presentation of results, the “ggplot2” package was adopted for visualization.

The number of articles and citations were applied as the bibliometric indicators. Briefly, productivity was measured by the number of publications (NP). The impact was measured by the number of total citations (TC) and average article citations per year (AC). H-index was used to predict future achievement and evaluate academic achievements by integrating productivity and impact. Besides, the latest impact factor (IF) based on the latest Journal Citation Reports was also chosen as an indicator reflecting the quality and impact of medical sources.

To comprehend the most highlighted keywords in the GAM field intuitively and quickly, a word cloud was constructed to extract the author's keywords. To deeply understand the categories and main information of the GAM field, a co-occurrence analysis of the author's keywords was carried out. Furthermore, the analysis of the most locally cited publications can be an important tool to measure the contribution of an article in a selected field, which may facilitate the researchers in finding innovative studies. Additionally, the trend topics analysis can help us to understand the research trends in the recent 20 years, thereby predicting future research hotspots.

## Results

### Main information of publications on GAM

**Figure 1:**
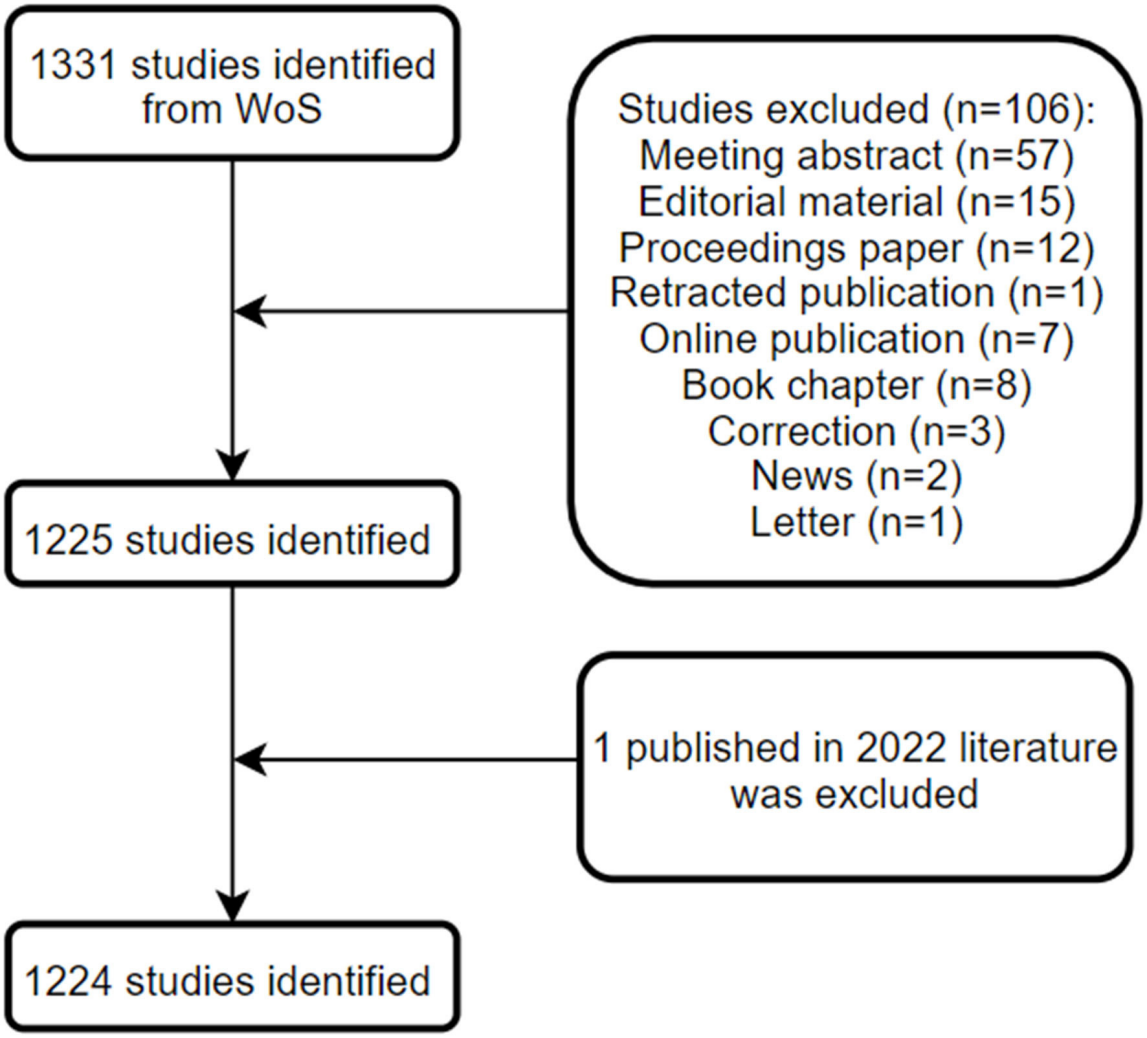
Flowchart of the screening process.

Until 16 January 2022, a total of 1,224 publications on GAM met our inclusion criteria and were eligible for subsequent bibliometric analyses, including 1,053 original articles and 171 reviews. The detailed process for screening and enrollment is presented in Figure 1. The average citations per publication were 33.8, with 4.8 of the average citations per year per publication. The main information of this collection can be found in Table 1.

**Table 1:** Main information of publications on GAM.

**Description**	**Results**
**Main information about data**	
Timespan	2002:2021
Sources (Journals, Books, etc)	403
Documents	1,224
Average years from publication	5.39
Average citations per documents	33.84
Average citations per year per doc	4.753
References	46,071
**Document types**	
Article	1,053
Review	171
**Document contents**	
Keywords plus (ID)	3,104
Author's keywords (DE)	2,551
**Authors**	
Authors	6,884
Author appearances	10,172
Authors of single-authored documents	7
Authors of multi-authored documents	6,877
**Authors collaboration**	
Single-authored documents	7
Documents per author	0.178
Authors per document	5.62
Co-Authors per documents	8.31
Collaboration index	5.65

### Analysis of annual publications on GAM

**Figure 2:**
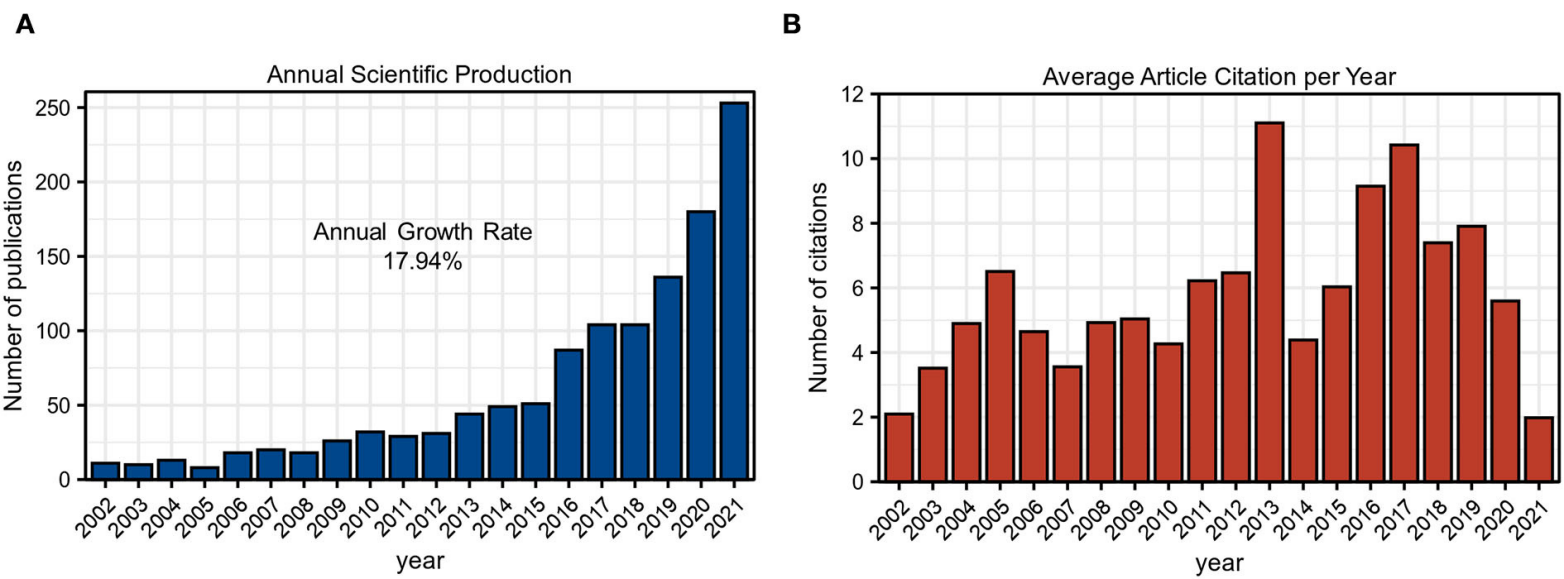
Annual publications analysis. **(A)** Annual scientific production of GAM research field as of 16 January 2022. **(B)** Average article citations per year of GAM research field of 16 January 2022.

Since 2002, the number of publications in the GAM field has elevated from 11 to 253 in 2021. As shown in Figure 2A and Table 2, the annual publications revealed gradual growth with an annual growth rate of 17.94% in the past 20 years. Figure 2B exhibits the average article citations per year, in which three peaks could be observed in 2005, 2013, and 2017, respectively. Combined with the wavy appearance, this index might encounter new peaks in the future. All of these results implied that GAM represented a hot research field and deserved consistent attention.

**Table 2:** The top 10 countries with the largest number of total citations.

**Country**	**TC**	**AC**	**NP**
USA	19,876	55.06	2,273
CHINA	5,545	16.36	1,916
GERMANY	4,054	45.55	651
JAPAN	1,886	37.72	237
ITALY	1,190	22.88	288
SWITZERLAND	968	69.14	104
POLAND	943	42.86	68
AUSTRALIA	760	30.40	145
FRANCE	632	21.07	204
NETHERLANDS	610	43.57	106

### Analysis of countries in publications on GAM

**Figure 3:**
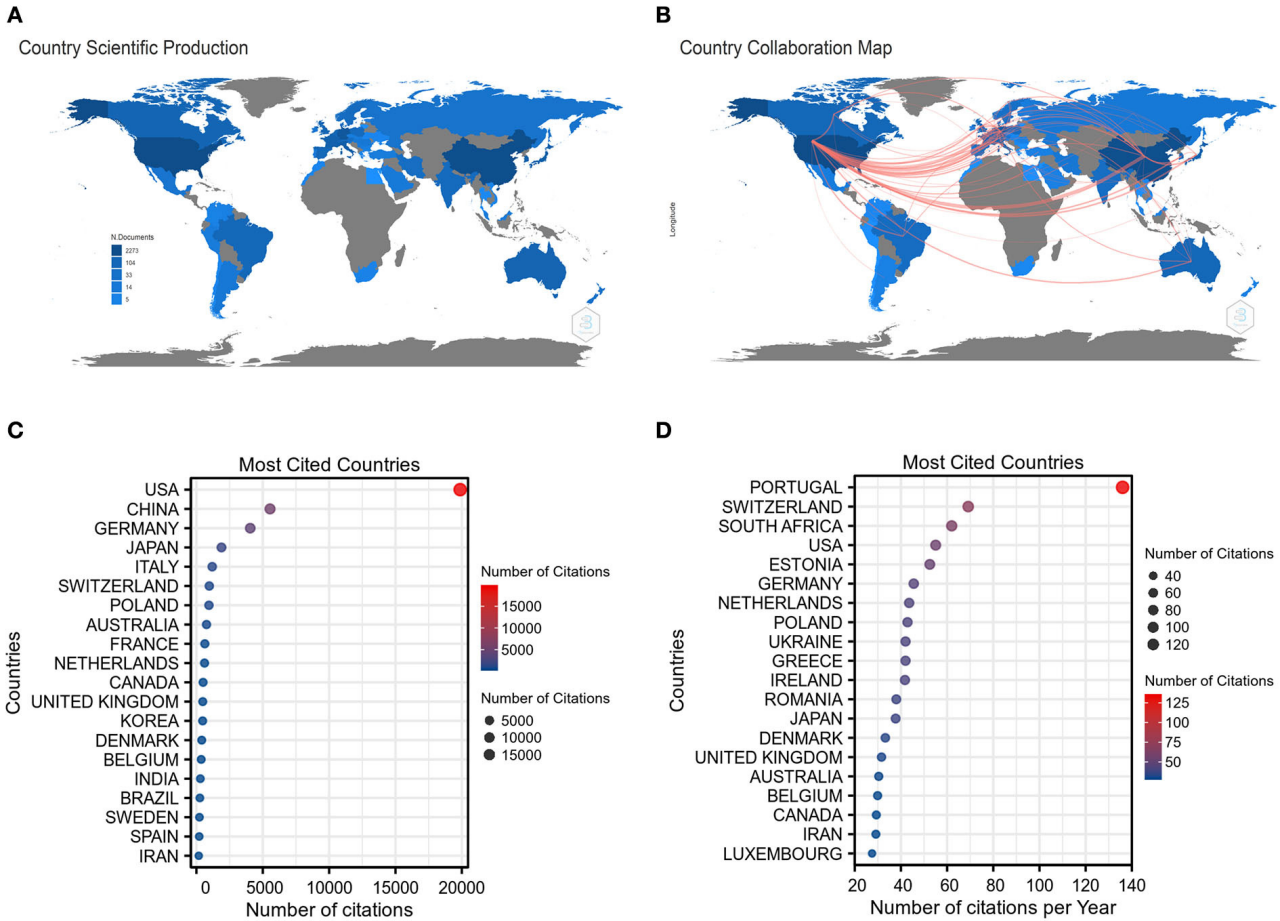
Countries analysis. **(A)** The world map of country scientific production in the GAM field. **(B)** The world map of country collaboration in the GAM field. **(C)** Top 20 most cited countries measured by the number of citations. **(D)** Top 20 most cited countries measured by the number of citations per year.

As depicted in Figure 3A, the corresponding authors in this collection were distributed in 57 countries. The intensity of blue on the world map reflects the NP of a chosen country and the more documents were published, the deeper the blue. Notably, the United States, China, and Germany had a deeper blue than any other countries, indicating that the three countries have the largest NP, accounting for 30.3, 25.6, and 8.7% of the total, respectively. Additionally, there were also several countries whose NP reached more than 1% of total publications, including Italy, Japan, South Korea, France, Australia, the United Kingdom, Canada, Netherlands, Switzerland, Spain, Brazil, and Belgium. Figure 3B exhibits the mapping of country collaborations in the GAM field, and the thickness of the red line represents the number of collaborations between countries. According to the thickness of red lines, the United States, China, and Germany appeared to be the core countries in the network. Of note, the collaboration between the United States and China has reached 68 times, which possessed the maximum thickness among all pairs. The amount of co-published papers between the United States and Germany attained 35 times.

The top 20 most cited countries measured by the number of citations are shown in Figure 3C. It is clear that the United States, China, and Germany remained the top-ranking countries regarding the TC. In particular, the TC of the United States was roughly 3.6 times that of China and ~4.9 times that of Germany. In terms of the AC, the publications from Portugal have more than 100 average article citations, which are much higher than that of any other country (Figure 3D). As the top three countries with respect to the NP, the average article citations of the United States, China, and Germany were not in the leading position, for which China merely had 16.36 average article citations.

### Analysis of authors in publications on GAM

In GAM-related publications, a total of 6,884 listed authors were retrieved for the subsequent analysis. Table 3 shows the results of the top 20 authors with the largest number of TCs. Among them, the number one author was Holland EC from the Fred Hutchinson Cancer Research Center, followed by Kettenmann from the Max Delbrück Center for Molecular Medicine and Washington University School of Medicine. The third top author was Gutmann DH from the Washington University School of Medicine. These top 20 authors contributed 174 publications, accounting for 14.2% of the total number of papers.

**Table 3:** The top 20 authors with the largest number of total citations.

**Authors**	**TC**	**H-index**	**NP**
Holland EC	1,848	8	9
Kettenmann H	1,828	13	13
Gutmann DH	1,772	15	21
Zhang J	1,657	14	24
Heimberger AB	1,523	10	15
Akkari L	1,520	4	4
Joyce JA	1,520	4	4
Wang Y	1,496	16	30
Huse JT	1,453	5	5
Quail DF	1,424	3	3
Brennan CW	1,379	2	2
Pyonteck SM	1,379	2	2
Sevenich L	1,379	2	2
Daniel D	1,354	2	2
Oei Y	1,354	2	2
Wei J	1,295	10	11
Hambardzumyan D	1,186	9	11
Wang Q	1,184	9	12
Leslie CS	1,141	1	1
Olson OC	1,141	1	1

### Analysis of sources in publications on GAM

As shown in Table 4, *Neuro-oncology* had the largest number regarding the TCs (2,228 citations), with *Cancer Research* (1,969 citations) and *Clinical Cancer Research* (1,812 citations) ranking second and third, respectively. Meanwhile, *Neuro-oncology* also shared the highest H-index (23) and NP (37 articles), implying that this journal had the most outstanding contribution to the publications on GAM. These top 20 sources outputted 311 publications, accounting for 25.4% of the total number of papers.

**Table 4:** The top 20 sources with the largest number of total citations.

**Sources**	**TC**	**H-index**	**NP**	**IF**
Neuro-Oncology	2,228	25	37	12.3
Cancer Research	1,969	21	27	12.7
Clinical Cancer Research	1,812	18	23	12.5
Glia	1,520	15	22	7.5
Cancer Cell	1,353	4	4	31.7
PLoS ONE	1,241	17	28	3.2
Proceedings of the National Academy of Sciences	1,149	10	12	9.6
Nature Medicine	1,141	1	1	53.4
Oncotarget	841	21	27	0
Oncoimmunology	827	14	17	8.1
Nature Neuroscience	788	3	3	24.9
Nature Communications	749	14	19	14.9
Journal of Pathology	687	6	6	8.0
International Journal of Cancer	580	14	15	7.4
Cell	547	3	3	41.6
Frontiers in Immunology	536	12	25	7.6
Oncogene	530	10	10	9.9
Experimental Neurology	521	2	2	5.3
American Journal of Pathology	519	7	8	4.3
Journal of Neuro-Oncology	489	12	22	4.1

### Analysis of keywords in publications on GAM

**Figure 4:**
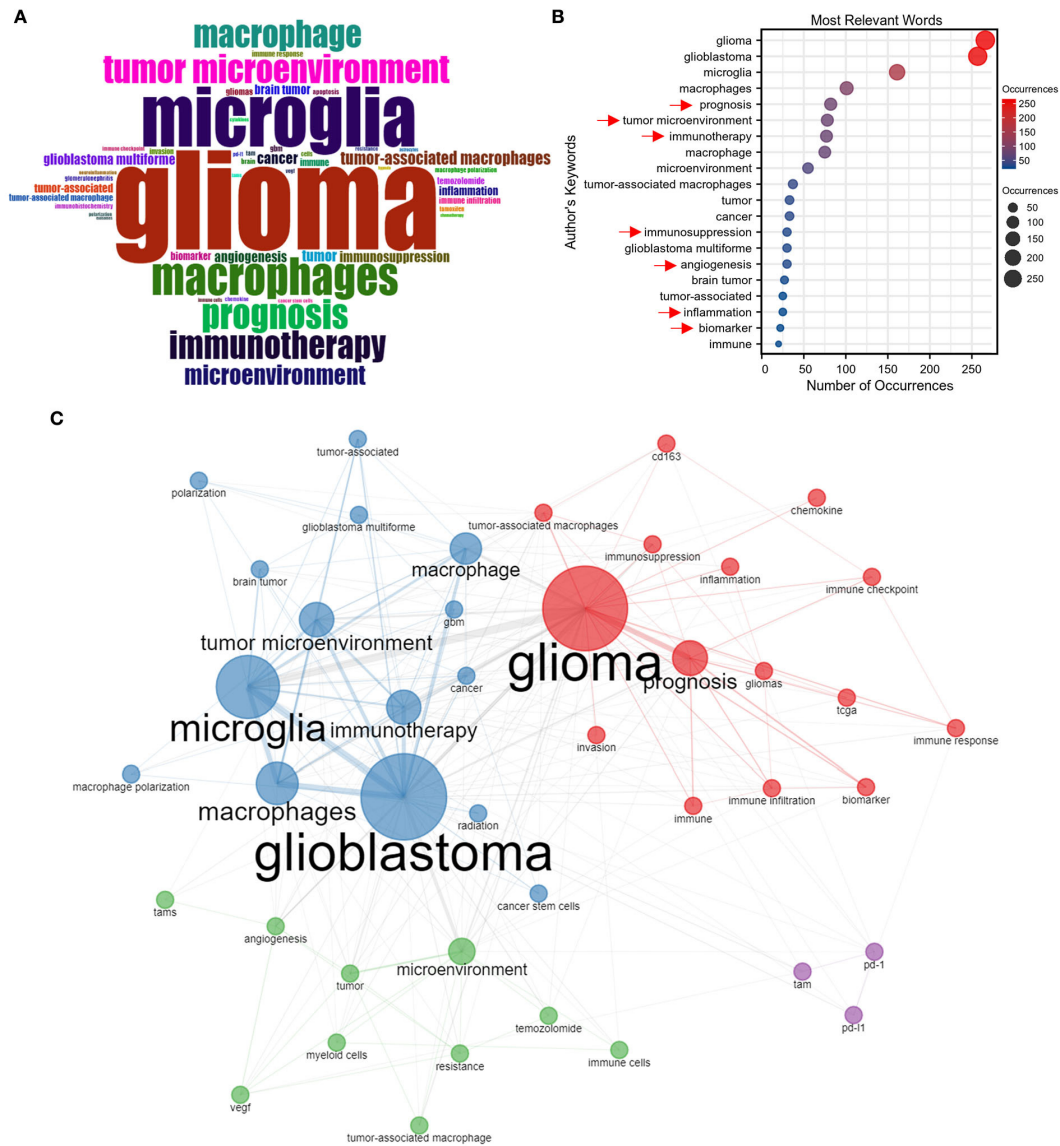
Keywords analysis. **(A)** World cloud of author's keywords in GAM articles measured by the frequency analysis. **(B)** Most relevant words measured by the number of occurrences. **(C)** Co-occurrence analysis of author's keywords.

Figure 4A maps the word cloud of keywords, and the font size of a word or phrase reflects the frequency of occurrences. The top 20 keywords are shown in Figure 4B. After excluding our searched terms, the most frequently appeared keywords in publications on GAM included “prognosis,” “tumor microenvironment,” “immunotherapy,” “immunosuppression,” “angiogenesis,” “inflammation,” “immune,” and “biomarker,” which might represent the research hotspot in GAM studies.

The co-occurrence network of keywords is summarized in Figure 4C, in which the keywords are divided into four color-coded clusters. The red cluster is mainly associated with the prognostic role of GAM and the identification of GAM-related biomarkers in reflecting the immune status of patients with glioma and predicting the efficacy of immunotherapies on the basis of the TCGA database. The cluster in blue focuses more on regimens that manipulated the state of GAM to reverse the immunosuppressive microenvironment of glioma, thereby improving the effectiveness of immunotherapies. The green cluster concentrates on the role of GAM in regulating the glioma microenvironment and the mechanism of GAM in promoting therapeutic resistance. The cluster in purple represents a relatively isolated cluster, yet it remains an important one, which included studies that focused on GAM-related PD-1 and PD-L1.

### Analysis of locally cited articles in publications on GAM

The top 10 most locally cited publications are presented in Table 5. The local citations of publications written by Hambardzumyan D in 2016 were 233, ranking first. In this review, the authors summarized the interaction between tumor-associated macrophage and glioma cells (24). In addition, a study conducted by Pyonteck et al. (25) proposed that tumor-associated macrophage could serve as a promising therapeutic target for proneural gliomas and demonstrated that CSF-1R inhibition might become a potential therapeutic strategy for patients with glioma. Meanwhile, Komohara et al. identified that the M2 macrophage marker (CD163) would be useful in predicting the prognosis for patients with glioma (13). Wu et al. discovered that cancer stem cells (CSCs) could mediate the shift of macrophages/microglia toward an immunosuppressive phenotype in glioma (23). Additionally, Gabrusiewicz et al. and Szulzewsky et al. focused on the unique phenotype of macrophage in glioma, which was different from the M1 or M2 subtype (26, 27). Anyway, these innovative studies have made outstanding contributions to the research field of GAM, facilitating the understanding of immunopathogenesis and the development of immune-adjuvant therapies.

**Table 5:** The top 10 most locally cited publications.

**Title**	**Corresponding author**	**Journal**	**Publication year**	**Local citations**	**IF**
The role of microglia and macrophages in glioma maintenance and progression	Kettenmann H	Nature neuroscience	2016	233	24.9
CSF-1R inhibition alters macrophage polarization and blocks glioma progression	Joyce JA	Nature medicine	2013	202	53.4
Possible involvement of the M2 anti-inflammatory macrophage phenotype in growth of human gliomas	Takeya M	Journal of pathology	2008	139	8.0
Glioma cancer stem cells induce immunosuppressive macrophages/microglia	Heimberger AB	Neuro-oncology	2010	102	12.3
Glioma-associated microglia/macrophages display an expression profile different from M1 and M2 polarization and highly express Gpnmb and Spp1	Kettenmann H	PLoS ONE	2015	89	3.2
Glioblastoma-infiltrated innate immune cells resemble M0 macrophage phenotype	Heimberger AB	JCI insight	2016	86	8.3
The molecular profile of microglia under the influence of glioma	Graeber MB	Neuro-oncology	2012	85	12.4
Microglia function in brain tumors	Badie B	Journal of neuroscience research	2005	84	4.2
Characteristics of the alternative phenotype of microglia/macrophages and its modulation in experimental gliomas	Kaminska B	PLoS ONE	2011	78	3.2
Microglial stimulation of glioblastoma invasion involves epidermal growth factor receptor (EGFR) and colony stimulating factor 1 receptor (CSF-1R) signaling	Segall JE	Molecular medicine	2012	77	6.4

### Analysis of trend topics in publications on GAM

**Figure 5:**
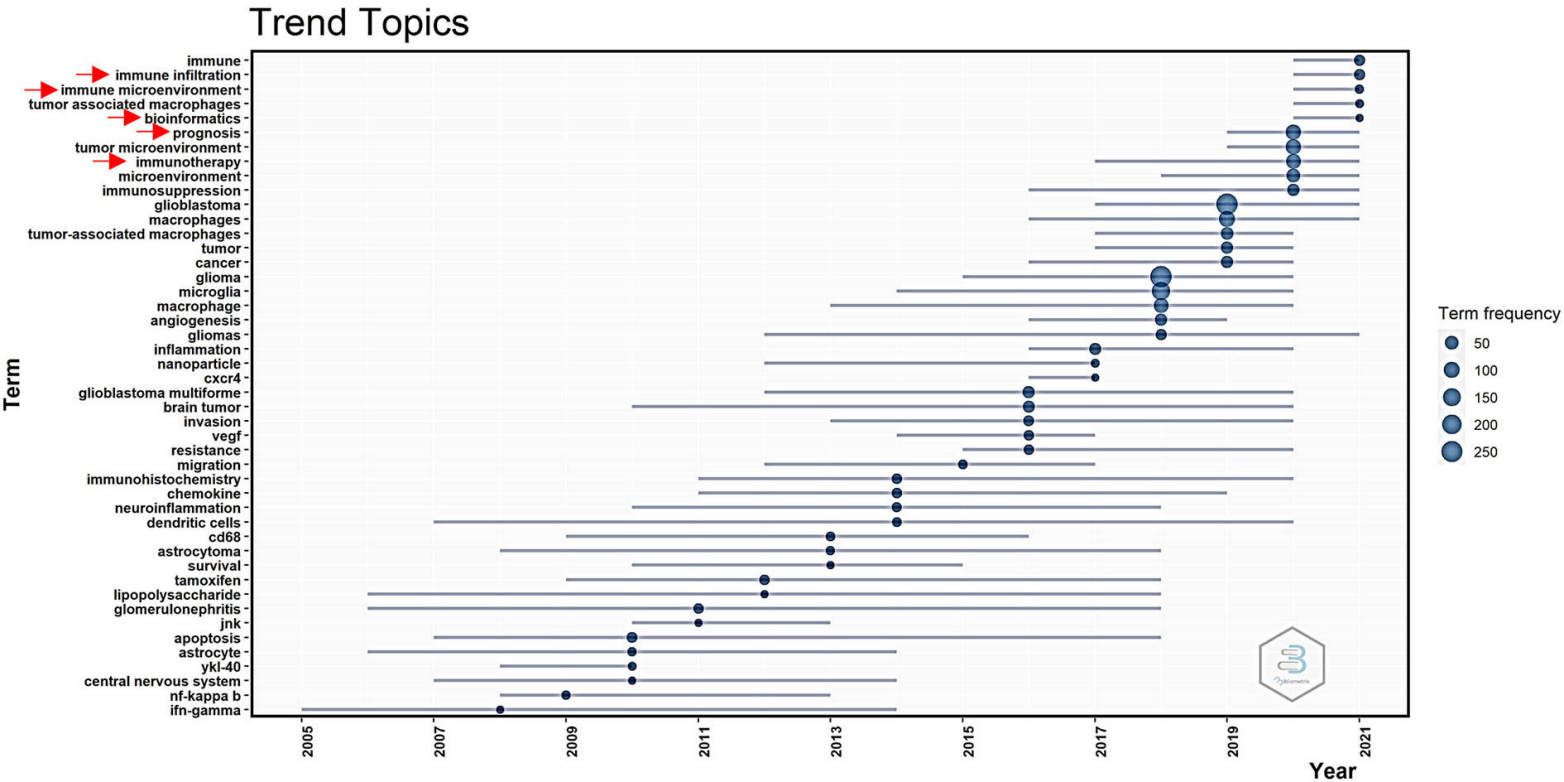
Trend topics measured by author's keywords.

The mapping of trendy topics revealed the “GAM” research tendency over time (Figure 5). Since 2017, attention has been given to “immunotherapy.” From 2019 to 2021, numerous novel topics on GAM were introduced, including “prognosis,” “bioinformatics,” “immune infiltration,” and “immune microenvironment.” The above topics deserved special attention in order to predict the coming hotspots in the research field of GAM.

## Discussion

In the current bibliometric analysis, we comprehensively mapped the current status, research hotspots, and tendency of publications on GAM using the R software with the “BiblioShiny” package. A total of 1,224 original articles and reviews published from 2002 to 2021 were retrieved for further investigation. According to the analysis of annual production, the number of publications revealed an overall upward trend (Figure 2A). The publications of most locally cited articles might be the main reason for three peaks in average article citations per year (Figure 2B). Overall, these findings implied that GAM gradually became the research focus in the scientific field and entered into a rapidly growing stage. Delineating publications and citations provided insight into the pattern of scientific production on the GAM.

According to the analysis of countries, the United States ranked first in terms of TC and NP, suggesting that it was a highly productive and influential country in GAM-related research (Table 2). However, when compared with the United States, the publications from China encountered a contradiction between quality and quantity, as evidenced by comparable NP but substantially lower TC. These results suggested that Chinese scholars should make more efforts on the quality of their studies, for which the application of more cutting-edge sequencing technologies might be helpful. Among the top 20 sources, 8 had relatively high IF (IF > 10). These results showed that it was possible to publish GAM-related studies in high-quality sources (Table 4). Notably, the top three sources (*Neuro-Oncology, Cancer Research*, and *Clinical Cancer Research*) have reached a balance between the yield and quality of studies on GAM. Paying attention to these top sources will facilitate us in grasping the frontiers of the research field of GAM, and publishing articles in these journals will contribute to the academic dissemination of our own research results. Of the top three authors in the GAM field, two are from the United States, and they all focused on the interaction between GAM and other components in the glioma microenvironment (24, 28–30). An in-depth understanding of the “cross-talk” mechanism will help to decipher the key features of the glioma immunosuppressive microenvironment, accelerating the development of GAM-related immune-adjuvant therapies and clinical transformation in this field.

As shown in the red cluster presented in Figure 4C, the “biomarker” and “prognosis” represented research hotspots in GAM studies. In earlier studies, the identification of GAM in glioma was mainly based on several markers, including CD163, CD204, and IL-10 (14, 15). However, with the development of monitoring approaches, scholars found that these markers representing GAM might not accurately reflect the real infiltering status. In recent years, as shown in Figure 5, bioinformatics methods based on transcriptome data in predicting immune infiltration have attracted more attention and became a hotspot. For example, CIBERSORT (31), TIMER (32), and xCell (33, 34) can quantitatively analyze the GAM in glioma tissue. These integrated methods could provide more information that could not be accomplished using single markers. By applying TCGA, CGGA, and the Rembrandt database, the association between GAM infiltration and clinical prognosis could be established. Zhang et al. constructed the microglia-related SubP28 signature that could precisely predict the prognosis for patients with glioma. In addition, based on the SubP28 signature, a comprehensive drug-subpathway network was established for identifying candidate drugs and feasible therapeutic targets (35). Therefore, recognizing glioma phenotypes and therapeutic responsiveness on the basis of GAM infiltration pattern and composition would be of prominent significance in the research field of GAM. Additionally, correlation analysis could be carried out to explore the cell–cell communications between GAM and the other components in the glioma microenvironment, providing a cellular and molecular basis for further investigation.

The heterogeneity of GAM in the glioma microenvironment also represented a research focus according to the blue cluster in Figure 4C. Some studies proposed that the majority of GAM displayed an M2-like phenotype, which played an immunosuppressive role that facilitated glioma progression in TME (7, 8, 14). Nevertheless, a study by Gabrusiewicz et al. revealed that GBM-infiltrated innate immune cells resemble M0 (undifferentiated) phenotype (26). In general, most of these studies were derived from *in vitro* experiments on cell lines such as THP-1 and RAW264.7. The THP-1 cell line was cultured from the blood of a boy with acute monocytic leukemia, and RAW264.7 represents a mouse leukemia cell line of monocyte (36–38). Upon stimulation, they could yield three phenotypes (M0, M1, and M2) for further analysis (39). Using cell lines is generally simple and risk-free, with a relatively rapid growth rate than that of primary cells. Moreover, the homogeneous genetic background further eliminates the degree of variability in studying macrophage phenotypes (38). Nonetheless, the disadvantages of using cell lines could not be neglected, for which the polarization of macrophage is distinct between human and mouse cell lines. More importantly, an *in vitro* study could not accurately reflect the actual functional status of GAM (2, 11). For example, unlike the primary culture of monocyte, THP-1 cells exhibit low levels of CD14, which plays an indispensable role in LPS signaling. Besides, the responsiveness of THP-1 cells upon stimulation has been reported to be lower than that of primary macrophages (40). Thus, more advanced models of *in vivo* experiments are an urgent need for exploring the heterogeneity of GAM. Meanwhile, emerging evidence has implied that GAM in glioma could not be simply divided into M1 and M2 subtypes, which came from *in vitro* experiments (41). In GBM tissues, GAM has been confirmed to develop a perplexed status that expressed both M1 and M2 markers (27). With the innovation of cutting-edge sequencing technologies, single-cell sequencing analysis and proteomics-based assay have been broadly applied in this field, which enabled researchers to decode significant heterogeneity within GAM. Müller et al. revealed that GAM possessed inherent heterogeneity. When compared with the microglial GAM, the blood-derived GAM had a unique phenotype that preferentially expressed immunosuppressive cytokines and exhibited an altered metabolic profile. They also pointed out that GAM-related therapies should focus on immunosuppressive blood-derived GAM but not target all GAM indiscriminately (42). Ochocka et al. demonstrated that GAM could be separated into three major groups, including microglia, infiltrating monocyte/macrophages, and border-associated macrophages. Additionally, these data uncovered a difference in GAM phenotype between males and females, for which significant upregulation of genes encoding MHCII was identified in microglia and infiltrating monocyte/macrophages of male mice. Further studies on GAM should take into consideration this discrepancy and avoid using single-sex research to speculate on the general population (43). Therefore, there is an urgent demand for a brand new classification system that can comprehensively yet accurately reflect the phenotypes of GAM.

The cross-talk between GAM and CSC is a topic of great concern (Figure 4C). Increasing evidence showed that CSC could regulate the recruitment, polarization, and survival of GAM in multiple manners. For example, CSC-derived IL-10 and IL-6 were demonstrated to potentiate a pro-tumor phenotype of GAM (23, 44). CSC can also enhance the infiltration of GAM through the production of OLFML3, POSTN, CCL5, CXCL1, and CXCL12B (7, 45–47). Furthermore, Tao et al. found that CSC could improve the survival of GAM *via* activating the α6β1 integrin/AKT pathway (8). Mirroring the function of CSC, GAM-derived factors were found to maintain the stemness of CSC (48). Thus, further discoveries will help identify the mechanism underlying their interaction, and targeting their interplay is expected to be an innovative therapeutic regimen for patients with glioma.

In recent years, immunotherapies-related studies have become a trendy topic according to Figure 5. However, T-cell-based immunotherapies have failed to induce an effective immunologic response in most patients with GBM (49), for which several reasons were responsible for these consequences. First, there is a preponderance of myeloid over lymphoid lineage in the glioma microenvironment, which is a unique feature of brain immunity in comparison with peripheral immunity (11). Massive infiltration of immunosuppressive GAM could jeopardize T-cell functions by expressing various co-inhibitory molecules and releasing inhibitory cytokines (50, 51). Second, since effector CD8^+^ T cells are rare in GBM tissues, they cannot mediate effective antitumor immune responses (52). Therefore, elucidation of the mechanism underlying immunosuppressive microenvironment remodeling by GAM might provide an important theoretical basis for the development of novel immunotherapeutic strategies against glioma. Recently, Chen et al. reported a cavity-injectable nanoporter-hydrogel superstructure that could generate glioma stem cells (GSCs)-specific chimeric antigen receptor (CAR) macrophage/microglia. Strikingly, these CAR–macrophage/microglia could target GSC and eliminate GSC by activating an adaptive antitumor immune response. Besides, they could also facilitate long-term antitumor immunity as they prevent postoperative glioma from relapsing in a mouse model (53). Future studies should focus more on novel therapeutic strategies underlying reprogramming GAM into exerting antitumor subtype and reversing immunosuppressive TME.

Similarly, the expression of PD-1 and PD-L1 on GAM has been a focus in this field, as presented by the purple cluster in Figure 4C. Wen et al. showed that the upregulation of PD-L1 was a remarkable feature of M2-like macrophage (54). Consistently, Gabrusiewicz et al. illustrated that CSC could drive differentiation of M2 macrophage and PD-L1 upregulation on human monocytes (55). By interacting with PD-L1 on GAM, PD-1 could drive an inhibitory signal in T cells, further attenuating the effector functions (5). Meanwhile, the expression level of PD-1 on macrophages was negatively correlated with phagocytic capacity (56). Notably, the blockade of PD-1 could substantially influence the phenotypical shift from M2 to M1 macrophage (57). Hence, regulation of the status of GAM using anti-PD-1/PD-L1 therapies is a research area that deserves further exploration.

Nonetheless, this study had several limitations. First, merely original articles and review articles written in English were included. Second, analysis based on the R package “BiblioShiny” might omit some information since it could not analyze the full text of enrolled publications. Third, only data obtained from the WoS database were included in this study; other databases need to be analyzed in a future study. Finally, the database updates continuously and only relevant records from 2002 to 2021 were taken into consideration in the current study. Therefore, a discrepancy might exist between the bibliometric analysis and the real status of publications on GAM.

## Conclusion

In conclusion, we performed an integrated bibliometric analysis of publications on GAM regarding different countries, authors, and sources, with the comprehensive mapping of the research hotspots over the past 20 years. Moreover, we also predicted the research trends of GAM-related studies. We hope our study can reflect the current status and novel directions for GAM research, thereby facilitating scholars to obtain more innovative research and rapid development in this field.

## Data availability statement

The original contributions presented in the study are included in the article/Supplementary material, further inquiries can be directed to the corresponding authors.

## Author contributions

Y-yL, R-qY, and L-yL conceived the bibliometric analysis. J-lL, Y-xL, and B-YT were responsible for the data interpretation. Y-yL and R-qY co-wrote the paper. H-yL and ZL undertook data collection. The final manuscript was approved by LC and Y-mY. All authors contributed to the article and approved the submitted version.

## References

[1] Lapointe S Perry A Butowski NA. Primary brain tumours in adults. Lancet. (2018) 392:432–46. 10.1016/S0140-6736(18)30990-5 30060998

[2] Wang H Xu T Huang Q Jin W Chen J. Immunotherapy for malignant glioma: current status and future directions. Trends Pharmacol Sci. (2020) 41:123–38. 10.1016/j.tips.2019.12.003 31973881

[3] Louis DN Perry A Wesseling P Brat DJ Cree IA Figarella-Branger D. The 2021 WHO classification of tumors of the central nervous system: a summary. Neuro Oncol. (2021) 23:1231–51. 10.1093/neuonc/noab106 34185076 PMC8328013

[4] Prados MD Chang SM Butowski N DeBoer R Parvataneni R Carliner H. Phase II study of erlotinib plus temozolomide during and after radiation therapy in patients with newly diagnosed glioblastoma multiforme or gliosarcoma. J Clin Oncol. (2009) 27:579–84. 10.1200/JCO.2008.18.9639 19075262 PMC2645859

[5] Chen Z Hambardzumyan D. Immune microenvironment in glioblastoma subtypes. Front Immunol. (2018) 9:1004. 10.3389/fimmu.2018.01004 29867979 PMC5951930

[6] Chen Z Zhuo S He G Tang J Hao W Gao WQ. Prognosis and immunotherapy significances of a cancer-associated fibroblasts-related gene signature in gliomas. Front Cell Dev Biol. (2021) 9:721897. 10.3389/fcell.2021.721897 34778248 PMC8586504

[7] Shi Y Ping YF Zhou W He ZC Chen C Bian BS. Tumour-associated macrophages secrete pleiotrophin to promote PTPRZ1 signalling in glioblastoma stem cells for tumour growth. Nat Commun. (2017) 8:15080. 10.1038/ncomms15080 28569747 PMC5461490

[8] Tao W Chu C Zhou W Huang Z Zhai K Fang X. Dual Role of WISP1 in maintaining glioma stem cells and tumor-supportive macrophages in glioblastoma. Nat Commun. (2020) 11:3015. 10.1038/s41467-020-16827-z 32541784 PMC7295765

[9] Charles NA Holland EC Gilbertson R Glass R Kettenmann H. The brain tumor microenvironment. Glia. (2012) 60:502–14. 10.1002/glia.21264 22379614

[10] Chen Z Feng X Herting CJ Garcia VA Nie K Pong WW. Cellular and molecular identity of tumor-associated macrophages in glioblastoma. Cancer Res. (2017) 77:2266–78. 10.1158/0008-5472.CAN-16-2310 28235764 PMC5741820

[11] Wei J Chen P Gupta P Ott M Zamler D Kassab C. Immune biology of glioma-associated macrophages and microglia: functional and therapeutic implications. Neuro Oncol. (2020) 22:180–94. 10.1093/neuonc/noz212 31679017 PMC7442334

[12] Liu Y Yao R Shi Y Liu Y Liu H Liu J. Identification of CD101 in glioma: a novel prognostic indicator expressed on M2 macrophages. Front Immunol. (2022) 13:845223. 10.3389/fimmu.2022.845223 35350788 PMC8957828

[13] Komohara Y Ohnishi K Kuratsu J Takeya M. Possible involvement of the M2 anti-inflammatory macrophage phenotype in growth of human gliomas. J Pathol. (2008) 216:15–24. 10.1002/path.2370 18553315

[14] Prosniak M Harshyne LA Andrews DW Kenyon LC Bedelbaeva K Apanasovich TV. Glioma grade is associated with the accumulation and activity of cells bearing M2 monocyte markers. Clin Cancer Res. (2013) 19:3776–86. 10.1158/1078-0432.CCR-12-1940 23741072

[15] Lu-Emerson C Snuderl M Kirkpatrick ND Goveia J Davidson C Huang Y. Increase in tumor-associated macrophages after antiangiogenic therapy is associated with poor survival among patients with recurrent glioblastoma. Neuro Oncol. (2013) 15:1079–87. 10.1093/neuonc/not082 23828240 PMC3714160

[16] Pesta B Fuerst J Kirkegaard EOW. Bibliometric keyword analysis across seventeen years (2000-2016) of intelligence articles. J Intell. (2018) 6:46. 10.3390/jintelligence6040046 31162473 PMC6480778

[17] Spreckelsen C Deserno TM Spitzer K. Visibility of medical informatics regarding bibliometric indices and databases. BMC Med Inform Decis Mak. (2011) 11:24. 10.1186/1472-6947-11-24 21496230 PMC3102604

[18] Zhang H Fan Y Wang R Feng W Chen J Deng P. Research trends and hotspots of high tibial osteotomy in two decades (from 2001 to 2020): a bibliometric analysis. J Orthop Surg Res. (2020) 15:512. 10.1186/s13018-020-01991-1 33168047 PMC7650161

[19] Rodrigues Sousa E Zoni E Karkampouna S La Manna F Gray PC De Menna M. A multidisciplinary review of the roles of cripto in the scientific literature through a bibliometric analysis of its biological roles. Cancers. (2020) 12:1480. 10.3390/cancers12061480 32517087 PMC7352664

[20] Akmal M Hasnain N Rehan A Iqbal U Hashmi S Fatima K. Glioblastome multiforme: a bibliometric analysis. World Neurosurg. (2020) 136:270–82. 10.1016/j.wneu.2020.01.027 31953095

[21] Nasir A Shaukat K Hameed IA Luo S Alam TM Iqbal F. Bibliometric analysis of corona pandemic in social sciences: a review of influential aspects and conceptual structure. IEEE Access. (2020) 8:133377–402. 10.1109/ACCESS.2020.3008733 34812340 PMC8545329

[22] Yao RQ Ren C Wang JN Wu GS Zhu XM Xia ZF. Publication trends of research on sepsis and host immune response during 1999-2019: a 20-year bibliometric analysis. Int J Biol Sci. (2020) 16:27–37. 10.7150/ijbs.37496 31892843 PMC6930382

[23] Wu A Wei J Kong LY Wang Y Priebe W Qiao W. Glioma cancer stem cells induce immunosuppressive macrophages/microglia. Neuro Oncol. (2010) 12:1113–25. 10.1093/neuonc/noq082 20667896 PMC3098021

[24] Hambardzumyan D Gutmann DH Kettenmann H. The role of microglia and macrophages in glioma maintenance and progression. Nat Neurosci. (2016) 19:20–7. 10.1038/nn.4185 26713745 PMC4876023

[25] Pyonteck SM Akkari L Schuhmacher AJ Bowman RL Sevenich L Quail DF. CSF-1R inhibition alters macrophage polarization and blocks glioma progression. Nat Med. (2013) 19:1264–72. 10.1038/nm.3337 24056773 PMC3840724

[26] Gabrusiewicz K Rodriguez B Wei J Hashimoto Y Healy LM Maiti SN. Glioblastoma-infiltrated innate immune cells resemble M0 macrophage phenotype. JCI Insight. (2016) 1:e85841. 10.1172/jci.insight.85841 26973881 PMC4784261

[27] Szulzewsky F Pelz A Feng X Synowitz M Markovic D Langmann T. Glioma-associated microglia/macrophages display an expression profile different from M1 and M2 polarization and highly express Gpnmb and Spp1. PLoS ONE. (2015) 10:e0116644. 10.1371/journal.pone.0116644 25658639 PMC4320099

[28] Wright-Jin EC Gutmann DH. Microglia as dynamic cellular mediators of brain function. Trends Mol Med. (2019) 25:967–79. 10.1016/j.molmed.2019.08.013 31597593 PMC6829057

[29] Ene CI Kreuser SA Jung M Zhang H Arora S White Moyes K. Anti-PD-L1 antibody direct activation of macrophages contributes to a radiation-induced abscopal response in glioblastoma. Neuro Oncol. (2020) 22:639–51. 10.1093/neuonc/noz226 31793634 PMC7229244

[30] De Boeck A Ahn BY D'Mello C Lun X Menon SV Alshehri MM. Glioma-derived IL-33 orchestrates an inflammatory brain tumor microenvironment that accelerates glioma progression. Nat Commun. (2020) 11:4997. 10.1038/s41467-020-18569-4 33020472 PMC7536425

[31] Ma W Zhang K Bao Z Jiang T Zhang Y. SAMD9 is relating with M2 macrophage and remarkable malignancy characters in low-grade glioma. Front Immunol. (2021) 12:659659. 10.3389/fimmu.2021.659659 33936093 PMC8085496

[32] Zhang M Wang X Chen X Zhang Q Hong J. Novel immune-related gene signature for risk stratification and prognosis of survival in lower-grade glioma. Front Genet. (2020) 11:363. 10.3389/fgene.2020.00363 32351547 PMC7174786

[33] Qi Y Deng G Xu P Zhang H Yuan F Geng R. HHLA2 is a novel prognostic predictor and potential therapeutic target in malignant glioma. Oncol Rep. (2019) 42:2309–22. 10.3892/or.2019.7343 31578594 PMC6826309

[34] Cai X Yuan F Zhu J Yang J Tang C Cong Z. Glioma-associated stromal cells stimulate glioma malignancy by regulating the tumor immune microenvironment. Front Oncol. (2021) 11:672928. 10.3389/fonc.2021.672928 33996602 PMC8117153

[35] Zhang C Zhao J Mi W Zhang Y Zhong X Tan G. Comprehensive analysis of microglia gene and subpathway signatures for glioma prognosis and drug screening: linking microglia to glioma. J Transl Med. (2022) 20:277. 10.1186/s12967-022-03475-8 35729639 PMC9210642

[36] Tsuchiya S Yamabe M Yamaguchi Y Kobayashi Y Konno T Tada K. Establishment and characterization of a human acute monocytic leukemia cell line (THP-1). Int J Cancer. (1980) 26:171–6. 10.1002/ijc.2910260208 6970727

[37] Chanput W Mes JJ Wichers HJ. THP-1 cell line: an *in vitro* cell model for immune *modulation* approach. Int Immunopharmacol. (2014) 23:37–45. 10.1016/j.intimp.2014.08.002 25130606

[38] Li P Hao Z Wu J Ma C Xu Y Li J. Comparative proteomic analysis of polarized human THP-1 and mouse RAW2647 macrophages. Front Immunol. (2021) 12:700009. 10.3389/fimmu.2021.700009 34267761 PMC8276023

[39] Shiratori H Feinweber C Luckhardt S Linke B Resch E Geisslinger G. THP-1 and human peripheral blood mononuclear cell-derived macrophages differ in their capacity to polarize *in vitro*. Mol Immunol. (2017) 88:58–68. 10.1016/j.molimm.2017.05.027 28600970

[40] Tedesco S De Majo F Kim J Trenti A Trevisi L Fadini GP. Convenience versus biological significance: are PMA-differentiated THP-1 cells a reliable substitute for blood-derived macrophages when studying *in vitro* polarization? Front Pharmacol. (2018) 9:71. 10.3389/fphar.2018.00071 29520230 PMC5826964

[41] Ransohoff RM. A polarizing question: do M1 and M2 microglia exist? Nat Neurosci. (2016) 19:987–91. 10.1038/nn.4338 27459405

[42] Müller S Kohanbash G Liu SJ Alvarado B Carrera D Bhaduri A. Single-cell profiling of human gliomas reveals macrophage ontogeny as a basis for regional differences in macrophage activation in the tumor microenvironment. Genome Biol. (2017) 18:234. 10.1186/s13059-017-1362-4 29262845 PMC5738907

[43] Ochocka N Segit P Walentynowicz KA Wojnicki K Cyranowski S Swatler J. Single-cell RNA sequencing reveals functional heterogeneity of glioma-associated brain macrophages. Nat Commun. (2021) 12:1151. 10.1038/s41467-021-21407-w 33608526 PMC7895824

[44] Yao Y Ye H Qi Z Mo L Yue Q Baral A. B7-H4(B7x)-mediated cross-talk between glioma-initiating cells and macrophages via the IL6/JAK/STAT3 pathway lead to poor prognosis in glioma patients. Clin Cancer Res. (2016) 22:2778–90. 10.1158/1078-0432.CCR-15-0858 27001312 PMC4891287

[45] Chen P Hsu WH Chang A Tan Z Lan Z Zhou A. Circadian regulator CLOCK recruits immune-suppressive microglia into the GBM tumor microenvironment. Cancer Discov. (2020) 10:371–81. 10.1158/2159-8290.CD-19-0400 31919052 PMC7058515

[46] Guo X Pan Y Gutmann DH. Genetic and genomic alterations differentially dictate low-grade glioma growth through cancer stem cell-specific chemokine recruitment of T cells and microglia. Neuro Oncol. (2019) 21:1250–62. 10.1093/neuonc/noz080 31111915 PMC6784288

[47] Chia K Mazzolini J Mione M Sieger D. Tumor initiating cells induce Cxcr4-mediated infiltration of pro-tumoral macrophages into the brain. eLife. (2018) 21: e31918. 10.7554/eLife.31918.022 29465400 PMC5821457

[48] Zhang X Chen L Dang WQ Cao MF Xiao JF Lv SQ. CCL8 secreted by tumor-associated macrophages promotes invasion and stemness of glioblastoma cells via ERK1/2 signaling. Lab Invest. (2020) 100:619–29. 10.1038/s41374-019-0345-3 31748682

[49] de Groot J Penas-Prado M Alfaro-Munoz K Hunter K Pei BL O'Brien B. Window-of-opportunity clinical trial of pembrolizumab in patients with recurrent glioblastoma reveals predominance of immune-suppressive macrophages. Neuro Oncol. (2020) 22:539–49. 10.1093/neuonc/noz185 31755915 PMC7158647

[50] Broekman ML Maas SLN Abels ER Mempel TR Krichevsky AM Breakefield XO. Multidimensional communication in the microenvirons of glioblastoma. Nat Rev Neurol. (2018) 14:482–95. 10.1038/s41582-018-0025-8 29985475 PMC6425928

[51] Gustafson MP Lin Y New KC Bulur PA O'Neill BP Gastineau DA. Systemic immune suppression in glioblastoma: the interplay between CD14+HLA-DRlo/neg monocytes, tumor factors, and dexamethasone. Neuro Oncol. (2010) 12:631–44. 10.1093/neuonc/noq001 20179016 PMC2940665

[52] Chongsathidkiet P Jackson C Koyama S Loebel F Cui X Farber SH. Sequestration of T cells in bone marrow in the setting of glioblastoma and other intracranial tumors. Nat Med. (2018) 24:1459–68. 10.1038/s41591-018-0135-2 30104766 PMC6129206

[53] Chen C Jing W Chen Y Wang G Abdalla M Gao L. Intracavity generation of glioma stem cell-specific CAR macrophages primes locoregional immunity for postoperative glioblastoma therapy. Sci Transl Med. (2022) 14:eabn1128. 10.1126/scitranslmed.abn1128 35921473

[54] Wen ZF Liu H Gao R Zhou M Ma J Zhang Y. Tumor cell-released autophagosomes (TRAPs) promote immunosuppression through induction of M2-like macrophages with increased expression of PD-L1. J Immunother Cancer. (2018) 6:151. 10.1186/s40425-018-0452-5 30563569 PMC6299637

[55] Gabrusiewicz K Li X Wei J Hashimoto Y Marisetty AL Ott M. Glioblastoma stem cell-derived exosomes induce M2 macrophages and PD-L1 expression on human monocytes. Oncoimmunology. (2018) 7:e1412909. 10.1080/2162402X.2017.1412909 29632728 PMC5889290

[56] Gordon SR Maute RL Dulken BW Hutter G George BM McCracken MN. PD-1 expression by tumour-associated macrophages inhibits phagocytosis and tumour immunity. Nature. (2017) 545:495–9. 10.1038/nature22396 28514441 PMC5931375

[57] Dhupkar P Gordon N Stewart J Kleinerman ES. Anti-PD-1 therapy redirects macrophages from an M2 to an M1 phenotype inducing regression of OS lung metastases. Cancer Med. (2018) 7:2654–64. 10.1002/cam4.1518 29733528 PMC6010882

